# Ultrasonographic data of cervical nerve roots diameter in 100 healthy adults

**DOI:** 10.1016/j.dib.2019.104776

**Published:** 2019-11-07

**Authors:** Miwako Kido, Yuji Hinode, Shugo Suwazono, Hiroyuki Akamine, Hiroshi Senoo, Naohisa Tatsuta, Yoshihisa Fujiwara, Ryo Nakachi

**Affiliations:** a Division of Neurology, National Hospital Organization Okinawa National Hospital, Japan; b Section for Laboratory Medicine, National Hospital Organization Okinawa National Hospital, Japan; c Center for Clinical Neuroscience, National Hospital Organization Okinawa National Hospital, Japan; d Division of Neurology, Ome Municipal General Hospital, Japan

**Keywords:** Sixth cervical nerve root, Nerve root diameter, Ultrasonography

## Abstract

Clinically significant evaluation of the diameters of nerve roots by ultrasonography requires the establishment of a normal reference range. Although there are multiple reports of nerve root diameters in normal subjects, none of them describe how to normalize and compare data derived from different facilities that may differ in their methodology, equipment, techniques, and recording sites during data acquisition. The aim of the present investigation was to establish a dataset of normal values using 100 healthy subjects, and to identify the factors that affect the normal ranges of cervical nerve root diameters with regard to age, sex, laterality, and root segments. Compared to previous reports, smaller standard deviations (0.07–0.21) were obtained, and the coefficient of variation ranged from 0.02 to 0.08, which facilitated the precise evaluation of cervical nerve roots. Age had a significant effect on the sixth cervical nerve root (C6) in male participants, and sex had a significant effect at C6 in participants in their 60s. To establish the normal values suitable for use across different facilities, acquired using different equipment, further development of various aspects, including the sophisticated recording techniques and data-sharing capabilities, is essential.

**Table:** Specifications Table

Subject area	Biology
Specific subject area	Biometry
Type of data	Table and Figure
How data were acquired	GE VividE9 ultrasound machine with 9 L linear array probe was used at 7–11 MHz
Data format	Raw
Experimental factors	Measurements were acquired from the neck by medical ultrasonography
Experimental features	Measurements were acquired at cervical C5, C6, and C7 levels and the following between–subject factors were recorded; age, sex, and laterality
Data source location	National Hospital Organization Okinawa National Hospital, Ginowan City, Okinawa, Japan
Data accessibility	accessible

**Table:** 

**Value of the Data** • The standard deviations (0.07–0.21) of the diameter of the cervical nerve root diameters in this study were smaller than that reported in previously • These data provide a basic and standard method for measuring the cervical nerve roots diameter using ultrasonography • These data are valuable for people who utilize ultrasonography data for cervical root evaluation, including doctors and laboratory technicians, and also are of benefit to patients with various diseases that affect cervical nerve roots

## 1 Data

**Fig. 1:**
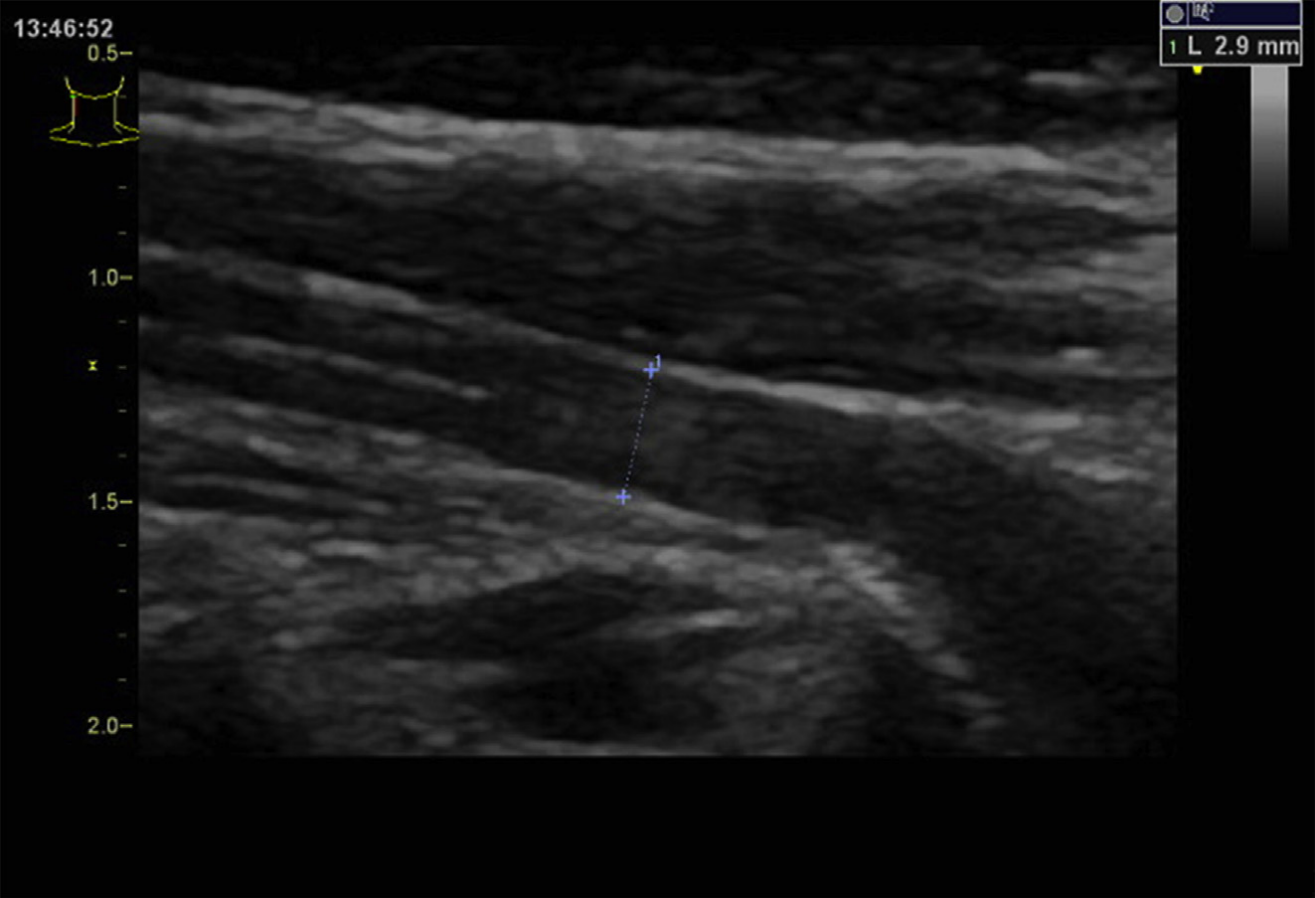
Example of the longitudinal image of the sixth cervical nerve root. The nerve root diameter was measured as the distance between the two cross marks.

The diameters of the cervical nerve roots are shown in Table 1, and a representative example of C6 nerve root used in this study is shown in Fig. 1. There were apparent differences in the diameters of the root segments;— C7 had the largest diameter, whereas C5 had the smallest (F = 4030, *p* < 0.00001). Laterality and sex of the participants did not affect the mean diameter (laterality, F = 3.88, *p* = 0.05; sex, F = 3.44, *p* = 0.07). In women, neither age nor laterality affected the mean diameters of the C5, C6, or C7 nerve root diameters (F = 1.03, *p* = 0.41). The diameter of the C6 nerve root was significantly affected by age only in men in their 20s (F = 3.23, *p* = 0.01) but not in those aged over 30 years (on multiple comparisons, the largest difference was obtained in the comparison between the 40s and 60s age group; however, the result was not significant; t = 1.47, *p* = 0.14). In subjects aged in their 30s and 60s, the mean nerve root diameter was significantly affected by sex (30s, F = 5.82, *p* = 0.02; 60s, F = 5.19, *p* = 0.02).

**Table 1:** Diameters of cervical nerve roots in mm.

	C5				C6				C7			
Age group (years)	MR	ML	FR	FL	MR	ML	FR	FL	MR	ML	FR	FL
20s	2.4	2.5	2.4	2.5	3.0	3.0	3.0	3.0	3.5	3.5	3.4	3.5
	0.10	0.09	0.11	0.07	0.11	0.12	0.07	0.13	0.08	0.11	0.12	0.06
	0.04	0.04	0.05	0.03	0.04	0.04	0.02	0.04	0.02	0.03	0.04	0.02
30s	2.5	2.5	2.4	2.5	3.1	3.1	3.0	3.0	3.4	3.5	3.5	3.5
	0.10	0.09	0.10	0.10	0.12	0.11	0.13	0.06	0.09	0.08	0.09	0.08
	0.04	0.04	0.04	0.04	0.04	0.04	0.04	0.01	0.03	0.02	0.03	0.02
40s	2.5	2.5	2.4	2.5	3.0	3.0	3.0	3.0	3.5	3.5	3.5	3.4
	0.09	0.09	0.18	0.09	0.10	0.11	0.13	0.13	0.08	0.07	0.10	0.08
	0.04	0.04	0.08	0.04	0.03	0.04	0.04	0.04	0.02	0.02	0.03	0.02
50s	2.4	2.5	2.5	2.5	3.0	3.0	3.1	3.1	3.5	3.5	3.5	3.5
	0.19	0.11	0.09	0.05	0.13	0.06	0.05	0.10	0.13	0.21	0.08	0.08
	0.08	0.04	0.04	0.02	0.04	0.02	0.02	0.03	0.04	0.06	0.02	0.02
60s	2.5	2.5	2.4	2.4	3.1	3.1	3.0	3.0	3.5	3.5	3.5	3.5
	0.11	0.09	0.08	0.06	0.09	0.08	0.05	0.09	0.06	0.06	0.14	0.10
	0.04	0.04	0.03	0.03	0.03	0.03	0.02	0.03	0.02	0.02	0.04	0.03

Top row, average diameter (mm); middle row, standard deviation; bottom row, coefficient of variation.

F, female; L, left; M, male; R, right.

## 2 Experimental design, materials, and methods

The participants were aged from 20 to 68 years; 10 men and 10 women were included in the age groups of 20s, 30s, 40s, 50s, and 60s. After thy signed informed consents, the diameters of the C5, C6, and C7 cervical nerve roots were measured on both sides in each subject by ultrasonography using a VividE9 system with a 7–11-MHz variable linear probe. Nerve root diameter was defined as the distance between one side of the epineurium and the other; the measurement was conducted at three points within 2 cm from the exit of the root. The average of these three measurements was taken as the final representative value of the nerve root, as previously described [1,2]. These representative values were assessed via analysis of variance, with two within-subject factors (side, right/left; level, C5/C6/C7) and two between-subject factors (age, sex) using ‘ANOVA4 on the Web’ [3]. This research was discussed and approved by the ethics committee of the National Organization Hospital Okinawa National Hospital.

## Conflict of Interest

The authors declare that they have no known competing financial interests or personal relationships that could have appeared to influence the work reported in this paper.

## References

[1] Takeuchi M., Wakao N., Kamiya M., Osuka K., Matsuo N., Terasawa T., Asai T., Takayasu M. (2014). Morphological distinction of cervical nerve roots associated with motor function in 219 healthy volunteers: a multicenter prospective study. Spine.

[2] Sugimoto T., Ochi K., Hosomi N., Mukai T., Ueno H., Takahashi T., Ohtsuki T., Kohriyama T., Matsumoto M. (2013). Ultrasonographic reference sizes of the median and ulnar nerves and the cervical nerve roots in healthy Japanese adults. Ultrasound Med. Biol..

[3] (2002). ANOVA 4 on the Web.

